# Achieving better outcomes for schizophrenia patients in Hong Kong: Strategies for improving treatment adherence

**DOI:** 10.1111/cns.13375

**Published:** 2021-02-08

**Authors:** William ak‐Lam Lo, Daniel Ki‐Yan Mak, Michael Ming‐Cheuk Wong, Oi‐Wah Chan, Eileena Mo‐Ching Chui, Dicky Wai‐Sau Chung, Glendy Suk‐Han Ip, Ka‐Shing Lau, Che‐Kin Lee, Jolene Mui, Ka‐Lok Tam, Samson Tse, Kwong‐Lui Wong, the Hong Kong Association of Psychosocial Rehabilitation (HKAPR)

**Affiliations:** ^1^ Kwai Chung Hospital Hong Kong China; ^2^ Private Practice Hong Kong China; ^3^ Department of Psychiatry Queen Mary Hospital Hong Kong China; ^4^ New Territories East Cluster (NTEC) Hong Kong China; ^5^ Department of Social Work and Social Administration The University of Hong Kong Hong Kong China; ^6^ Hong Kong Society of Professional Training Hong Kong China; ^7^ Department of Psychiatry The Chinese University of Hong Kong Hong Kong China; ^8^ Department of Psychiatry Castle Peak Hospital Hong Kong China; ^9^ Social Worker Hong Kong China

**Keywords:** antipsychotic agents, Hong Kong, paliperidone palmitate, patient compliance, schizophrenia

## Abstract

Recent research on second‐generation long‐acting injectable antipsychotics (LAI SGAs) has proven its effectiveness in minimizing medication nonadherence problem and reducing relapses. Administered by medical professionals, making quick detection of nonadherence possible, long‐acting injectable antipsychotics (LAIs) facilitate immediate intervention and recovery process, and thus are favored by psychiatrists. Despite a higher initial cost with LAIs, the subsequent schizophrenia‐related health costs for hospitalizations and outpatients are greatly reduced. With reference to guidelines published by psychiatric associations around the globe, this article looks at scenarios in Hong Kong on the management of severe mentally ill patients with regard to the use of a host of psychosocial interventions as well as LAI SGAs as a preferable treatment. In particular, it examines the benefits of using LAI SGAs for Hong Kong patients who demonstrated high nonadherence treatment rates due to their social environment. It assesses the rationale behind the early usages of LAI SGAs, which help to provide better recovery outcomes for patients.

## 1 INTRODUCTION

In Hong Kong, there are more than 40 000 diagnosed schizophrenia patients, of which around half will be provided with outreach support in the community over the next few years.^1^ Health Authority (HA), as a major specialist service provider for people with mental disorders in Hong Kong, provides psychiatric services ranging from inpatient facilities, day hospitals, and specialist outpatient (SOP) clinics to community outreach services. Among these services, SOP clinics serve the largest proportion of patients within the population (89%).^1^ The clinics had served 26 747 new patients in 2008‐09 and provided a total of 647 864 outpatient attendances in the same year. Moreover, the workload in these clinics has increased by 19% since 2003‐04.^1^ The increase in the burden of mental illness suggests that the HA will need to enhance its SOP and community services for rehabilitation further to accommodate the rising number of utilizations. In addition, the support of mental health promotion, including prevention, early detection, and treatment, are important in maintaining a healthy society.^1^, ^2^, ^3^

Although regular intake of medication is the key to preventing relapse for patients with mental illness, treatment nonadherence and relapse remain the major problems for many schizophrenia patients,^4^ indicating that there is a need to improve severe mental illness (SMI) management for better long‐term treatment outcomes.

Meanwhile, advances in psychosocial and pharmacological interventions continue to improve the management of schizophrenia.^5^, ^6^, ^7^ Second‐generation antipsychotics (SGAs) have become the foundational interventions for schizophrenia as they offer good treatment efficacy with superior tolerability relative to first‐generation antipsychotics (FGAs), especially in diminishing the development of extrapyramidal side effects.^8^ Because of this advantage and other reasons, for instance, the once biweekly or monthly injection schedule, SGA long‐acting injectable antipsychotics (LAIs) further minimize the medication nonadherence problem, which often results in relapses and poses as a major barrier to optimal recovery.

A recent study revealed that 41% of Hong Kong psychiatrists would prefer to switch to or add on a LAI.^4^ Despite such preferences, the use of LAI is restricted due to resource limitations and stringent SMI management (eg, for the year 2011, 5.5% of Hong Kong's GDP was spent on health care, but only 0.24% was distributed to mental health).^9^

The aim of this article is to provide directions for Hong Kong on improving the SMI management for schizophrenia patients in the near future. The advantages and preferences of LAI SGAs over FGAs and current issues related to the management of SMI are discussed.

## 2 METHODS

An expert panel from the Hong Kong Association of Psychosocial Rehabilitation (HKAPR) was formed to study on current issues related to the management of schizophrenia. Based on the scientific evidence in the literature and the clinical experience encountered by the healthcare professionals, recommendations for the improvement of SMI management were provided in the hope of achieving better treatment outcomes for patients.

A systematic literature search was performed using the PubMed database with the keywords “schizophrenia”, “first‐generation antipsychotics”, “second‐generation antipsychotics”, “long‐acting injectable antipsychotics”, “oral antipsychotics”, “serious mental illness” and “medication adherence”. Approximately 1000 articles published from January 1981 to June 2016 resulted from the searches. A total of 61 articles and relevant references cited in those articles were selected for further review. In addition, the Hospital Authority Mental Health Service Plan for Adults 2010‐2015^1^ was included. All articles selected in this article were published in English.

### 2.1 Impact of schizophrenia and better treatment outcomes on the society

**FIGURE 1:**
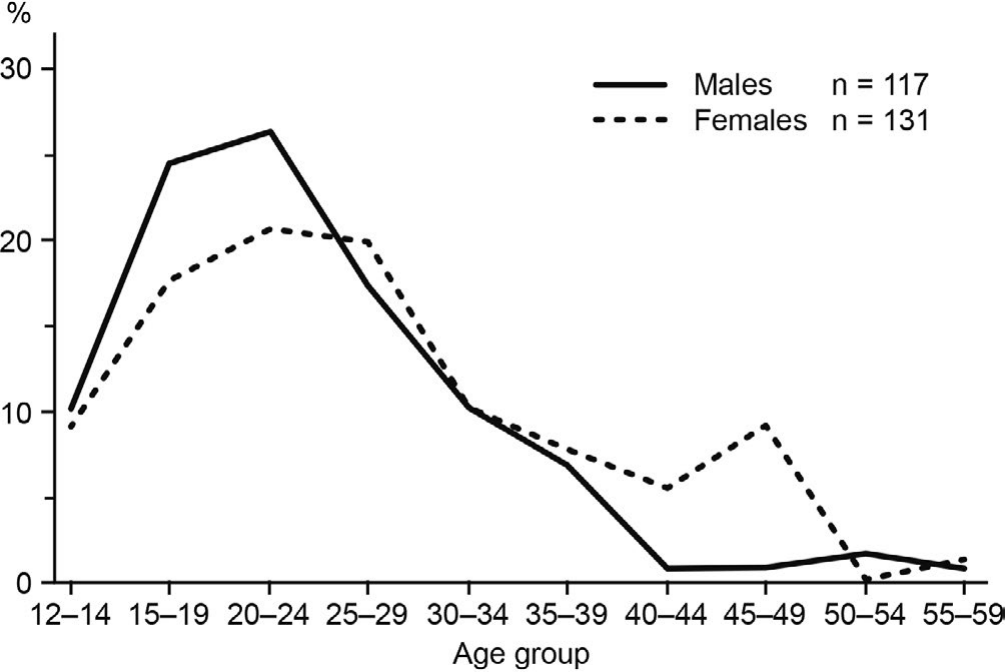
Age distribution of onset of schizophrenia (first sign of mental disorder) for men and women. From Häfner et al, 1998^10^

The onset of schizophrenia peaks between the age of 20 and 24 in men, and between the age of 25 and 29 in women, with another small peak after age 45 (Figure 1).^10^ Patients with schizophrenia often deny their mental problems; however, the disease progressively causes deterioration in their daily life functioning, such as occupation, hygiene, self‐management, and their family and social relationships. All these negatively impact on the patients’ quality of life (QoL) and the people around them.^11^ In addition to the deterioration, sudden and unexpected personality changes, social stigmatization, and discrimination from other people could make patients less adherent to treatments and hence more prone to relapses.^11^

Generally, if longer remission can be achieved and relapses can be prevented early in the course of the illness, the brain tissue loss and the decline in functioning can be minimized.^12^, ^13^ This would increase the chance that the patients will reintegrate into the society and allow for more opportunities for patients to rebuild relationships and regain functions early.

### 2.2 Voices, concerns, and myths from patients

A study conducted by Caroli et al^14^ found that 39% of patients with schizophrenia did not believe they had mental illness. Only 28% realized that they were diagnosed with schizophrenia. Indeed, the name of their disease was unknown to 37% of the patients, and 35% believed that their illness was mainly other psychological problems such as depression.^14^ Moreover, patients with schizophrenia who received an injectable treatment were concerned with the details of the injection procedures. 37% of the patients stated that they were unable to recall being given any details on the advantages and possible side effects of the injectable treatment.^14^ Additionally, Potkin et al^15^ showed that patients refused to receive the injectable treatment mainly due to fear and were concerned with the damaging and side effects of the therapy.^15^

For patients in Hong Kong, a cohort study revealed that patients’ symptom severity, psychosocial functioning, self‐esteem, and duration of illness were associated with their own perceived stigmatization.^16^ Those who experienced stigmatization was mainly due to their inadequate knowledge of mental illness, ineffective communication, and misunderstandings about the disease mostly learnt from their relatives and the mass media, thus resulting in a feeling of inferiority and more stress for the patients. The study also showed that the perceived stigmatization from patients significantly increased over 1 year with deteriorated self‐esteem and mental state. These perceptions can, in turn, increase the patients’ relapse rates.^16^

To address the patients’ concerns and promote advanced recovery among people with SMI, shared decision‐making (SDM) should be implemented. Patients with schizophrenia may wish to be involved in decisions about their treatment.^14^ In a recovery‐oriented mental healthcare system, instead of just focusing on interventions that reduce symptoms, the process should be directed to the conceptualization of personal recovery in mental health (ie, the CHIME framework: Connectedness, Hope, and Optimism about the future, Identity, Meaning in life, and Empowerment).^17^, ^18^ Interventions that target these recovery outcomes, such as the illness management and recovery program (IMR), can be implemented to teach illness self‐management strategies to people with SMI.^18^ To overcome the ethical and cultural challenges of implementing SDM, two approaches are suggested: (a) social marketing: a citizen‐centered mental health system oriented around the preferences of service users, in which partnership is the foundation rather than an add‐on feature; (b) hospitality industry: an approach where clinicians work in partnership with service users to ensure that they have a positive experience.^19^

### 2.3 Voices, concerns, and myths from family and caregivers

A study conducted with the caregivers from Hong Kong, who had family members with mental illness, showed that their QoL was significantly lower than that of other Chinese populations.^20^ Results indicated that caregivers who were younger had chronic illness, and a lower level of education and more dissatisfaction with mental health services were associated with more burdens and poorer QoL. Particularly, caregiver characteristics and beliefs were strongly associated with caregiver's QoL.^20^

Indeed, the insight, concerns, and characteristics of the family members have a great influence on the patients’ treatment adherence. The environmental and cultural contexts of the clinical setting and family dynamic are important areas to consider when using medication interventions in Hong Kong.^21^ For example, some family members do not believe in Western Medicine, or prefer Traditional Chinese Medical practices to Western Medicine.^21^ The traditional views of mental illness held by some families/carers may present a barrier to achieving treatment adherence, suggesting that in Hong Kong it may be beneficial to modify medication training to include specific family work approaches aimed to explore the cultural beliefs of mental illness and its management.^21^

Moreover, the distorted perspectives of some caregivers may present barriers to using psychotropic medications, including the recent LAIs.^22^ According to a research aimed to study the perspectives of healthcare providers (psychiatrists, nurses, social workers, and therapists) about LAIs in the United States, caregivers were mostly concerned about potentially damaging the therapeutic relationship and possible side effects when discussing about initiating LAIs.^15^ A study of community mental health nurses in the United Kingdom revealed that about one‐third of the nurses believed that LAIs were old‐fashioned, about 40% felt that LAIs were stigmatizing and coercive, and about one‐quarter of those surveyed felt that LAIs compromised patient autonomy.^23^

Despite these concerns, the majority of healthcare providers (especially those in the mental health professions) had positive attitudes toward LAIs.^15^, ^24^, ^25^, ^26^ In a study of 4120 nurses from Europe, Middle East, and Africa, almost all respondents (92%) felt that ensuring continuous medication with an LAI antipsychotic would yield long‐term patient benefits.^25^ Similarly, based on a study conducted to assess the psychiatric nurses’ attitudes to depots in Hong Kong, 82.7% agreed that patient's adherence is better with depots than with oral antipsychotics (AP).^24^

### 2.4 Standard treatment algorithm and patient journey to recovery

The classical treatment goal for treating schizophrenia included symptom reduction and control, preventing relapses and increasing adaptive functioning so that the patient can be reintegrated into the community.^26^ Pharmacotherapy is the mainstay of schizophrenia management. Nonetheless, the very first step of treatment should be a detailed and thorough assessment and an accurate diagnosis for schizophrenia followed by appropriate pharmacological treatment.^27^ The international guidelines (such as the American Psychiatric Association and the Maudsley Prescribing Guidelines) suggested trial of a single SGA for first episode of schizophrenia.

Although there are no strict local guidelines for the treatment duration of schizophrenia, psychiatrists should judge on a case‐by‐case basis. Patients who previously remitted while on medications for a continuous period of time cannot be warranted to be in full remission if the antipsychotics were stopped.^28^ For patients with first‐episode psychosis (FEP), AP treatment is often discontinued after 1 year of good response. However, a study conducted in Hong Kong demonstrated that patients with FEP who were given placebo in the following year had a much higher rate of relapse than those who received maintenance treatment, suggesting the importance of treatment continuity.^27^, ^29^

Furthermore, in the past two decades, the traditional hospital‐based care in Hong Kong has evolved toward community‐based care for mental patients, including those who are suffering from schizophrenia. To further enhance the support to patients discharged from hospital, a proactive and assertive model of community services adopting the case management approach was started in 2010. In addition to the conventional community psychiatric nursing service (CPNS), three rehabilitation programs including Intensive Care Team (ICT), Personalized Care Program (PCP), and Community Psychiatric Service (CPS) were implemented. Standardized protocols are followed, and services are provided after assessing patients’ risks and needs. The case managers can be nurses, occupational therapists, or trained social workers. They work closely with the carers, psychiatrists and other mental health professionals, nongovernmental organizations (NGOs), and other rehabilitation agencies that provide services to patients. This community treatment model allows intensive psychosocial interventions for patients with schizophrenia to be carried out in the community and optimized their treatment outcomes and recovery.^30^

### 2.5 Clinical and social importance of treatment adherence

Nonadherence is a vital problem in treating schizophrenia patients, which has been reported to be as high as 50% with the use of oral AP.^31^, ^32^ In Hong Kong, the self‐reported nonadherence rate was 30%.^21^ Because nonadherence can lead to a high risk of relapse with subsequent clinical and social implications,^27^ a phase‐specific review of treatment regimen should be conducted regularly.

The negative effects of relapse on brain tissues have been well documented in several studies.^12^, ^13^, ^33^ Brain tissue loss has been linked to functional significance, which is related to the severity of the psychotic symptoms and cognitive impairment,^13^ implying that frequent relapses will worsen the patient's chance on achieving normal daily life. Additionally, there is an increased risk of hospitalization due to relapses, which will further contribute as social burden on patients, their families, and the society. It may also increase the safety concerns on the society due to an increased risk of dangerous behaviors such as suicide or homicide if the mental state was unstable.^12^, ^13^, ^27^, ^33^

Indeed, the chance of relapse is increased if pharmacotherapy or illness management plan discontinues, and with each relapse, recovery can be slowed, and the course of illness worsened.^34^, ^35^, ^36^, ^37^ A study conducted by Robinson et al^38^ showed that FEP patients who discontinued treatment were almost five times more likely to experience relapse than adherent patients. Also, the illness became more resistant to treatment and more difficult for patients to achieve functional recovery.^38^ Additionally, a 15‐year prospective study from the Netherlands revealed that the chronicity of psychotic symptoms for patients gradually increased from 27% to 47% between the first and the fourth episodes, and relapses occurred with a high risk of suicide.^39^ The data from these studies clearly support the need for an adequate relapse prevention program for mentally ill patients.

### 2.6 Assessing drug adherence

In Hong Kong, the patient's medication adherence is mainly assessed indirectly. For example, counting the number of pills that remain in the patient's medication bottles or vials is a common method for measuring adherence.^32^, ^34^ However, it can be a barrier to build rapport with the patient because the patient may feel that she/he is being pressured or not trusted by the healthcare workers. Furthermore, the patient can still discard some of the pills before hospital visits to pretend that she/he is following the treatment regimen. Besides checking the serum drug levels, there is no accurate way of assessing the medication adherence, and specific serum drug testing may be burdensome and impractical for routine clinical services.^27^, ^34^ Nevertheless, there are a few commonly used adherence rating scales for the assessment of drug adherence including the Drug Attitude Inventory (DAI),^40^, ^41^ the Medication Adherence Rating Scale (MARS),^41^ and the Brief Adherence Rating Scale (BARS).^42^ The DAI is a commonly used instrument that contains 10/30 items focusing on subjective attitudes toward antipsychotics; the MARS is a 10‐item self‐reported inventory which is based on the DAI; and the BARS is a clinician‐administered adherence instrument. These rating scales demonstrate good sensitivity and specificity in identifying nonadherent outpatients.^27^

One way to improve medication adherence is to adopt a *concordance approach* in introducing treatment options to the patients. The term “concordance” relates to a consultation process in which prescription is based on partnership because patients are more likely to be committed to the treatment regimen and adhere to the decision that she/he has actively made for herself/himself.^43^ Concordance between the clinician and the patient should be the goal of all SDM encounters.^44^ Three key steps to achieving SDM in clinical practice are as follows: (a) Choice talk, referring to the step of making sure that patients know that reasonable options are available, (b) Option talk, referring to providing more detailed information about options, and (c) Decision talk, referring to supporting the work of considering preferences and deciding what is best.^45^

### 2.7 Use of LAIs to address the adherence problem

Survey results published by the South China Morning Post (SCMP) stated that people in Hong Kong who work long and inflexible hours are more vulnerable to mental illnesses.^46^, ^47^ Long working hours, as well as the lack of private working spaces for most of the workers, can be very troublesome for patients to strictly adhere to taking pills several times a day. Moreover, patients will also have to deal with the stigma or enquiries regarding the medication from their coworkers. These factors will contribute to patients’ nonadherence to the oral drugs.

Most of the frequent causes of treatment failure are due to adherence problems.^48^, ^49^ Patients will initially prefer to take oral drugs, but as their symptoms subside, they often would reduce the amount of drugs taken on their own. Patients may also stop treatment at the first sign of side effect that causes inconvenience.^50^

Depending on the specific drug, LAI SGAs only need to be administered once every 2‐4 weeks. Additionally, LAIs can be used to address the adherence issues. The treatment not only decreases the patient's burden of daily pill‐taking, but also alerts the healthcare workers of patient's nonadherence as LAIs are administered by healthcare workers directly.^22^ Compared with oral AP, the use of a LAI SGA agent was associated with better QoL outcomes and lower caregiver burden.^51^ Initially, patients might be reluctant for LAIs, but as they are stabilized over the long‐term treatment, their attitude may change. By lowering the caregiver's burden on checking patients’ adherence to daily pill‐taking, the potential conflict between them may also be reduced.

Compared with the oral medication, LAIs have several advantages. Firstly, they are administered by mental health professionals that have the potential of increasing therapeutic contacts. This contact allows for easy and quick detection of nonadherence which facilitates immediate intervention. Secondly, it decreases the risk of accidental or deliberate overdose. Thirdly, the parenteral route avoids first‐pass metabolism in the liver, which reduces the risk of drug‐drug interactions. Last but not least, injections offer stable plasma concentrations that avoid high fluctuations and reduce the risk of drug levels wavering below or above the desired range.^52^

**FIGURE 2:**
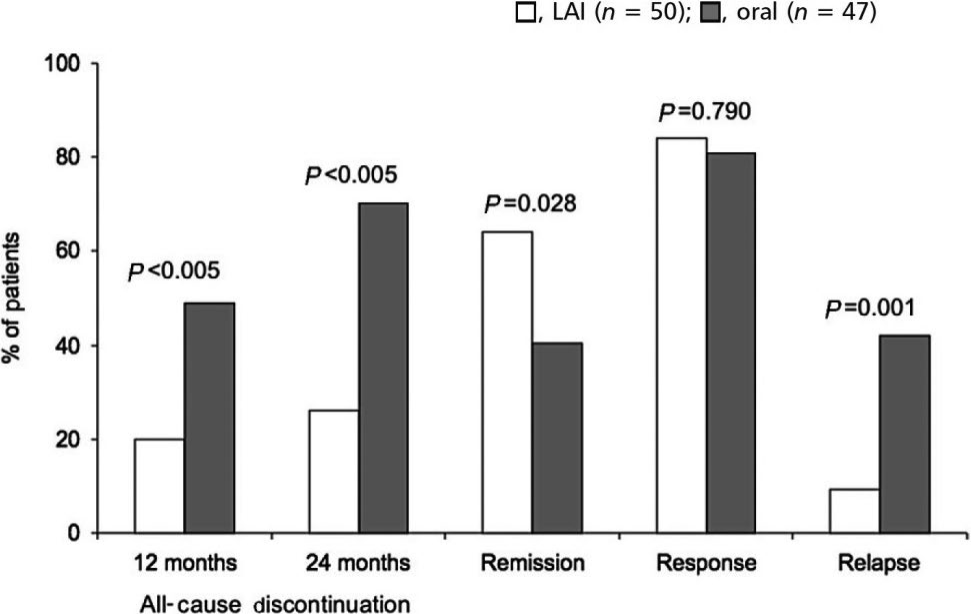
Outcomes after treatment with LAI or oral antipsychotics. From Stevens et al, 2016^22^

Long‐acting injectable antipsychotics are known to be at least as effective as oral AP; however, LAIs are associated with a significantly lower percentage of relapse and treatment discontinuation, with a significantly higher percentage of remission (Figure 2).^22^ Based on a hospital database analysis for 4 years, LAI SGAs were also associated with lesser rehospitalization and emergency room visits compared with oral AP.^53^

### 2.8 Early interventions with LAIs

Empowerment and interpersonal support are important components in the recovery process for schizophrenia patients.^54^ Moreover, both pharmacological and psychosocial treatments should be offered as early interventions, as these therapies can improve patients’ prognosis and help prevent their illness chronicity.^55^ Several rationales for using LAIs early in the treatment of schizophrenia are as follows^22^: (a) neuroprotective potential: LAIs promote intracortical myelination and may exert a neuroprotective effect when used in patients with FEP; (b) relapse reduction: A significantly lower relapse rate was observed with the LAIs; and (c) Adherence and overall illness management were easily fostered with the LAIs. The three most commonly used modalities of psychosocial interventions, namely, cognitive behavioral therapy (CBT), family intervention, and psychoeducation, also demonstrated a significant reduction of relapse during a 12‐month follow‐up study.^55^ Compared to medication alone, an integrated treatment approach consisting of pharmacotherapy, psychosocial treatment, and psychoeducation also resulted in higher medication adherence, suggesting that this approach could effectively help patients learn the necessary skills about symptoms and medication management, which, in turn, could help prevent relapse.^56^

Indeed, several professional associations have issued guidelines with recommendation of the early use of LAIs. In 2009, the National Institute for Health and Clinical Excellence (NICE) guidelines stated that clinicians should consider offering depot/LAI antipsychotic medications to patients with schizophrenia who would “*prefer such treatment after an acute episode and where avoiding covert nonadherence to antipsychotic medication is a clinical priority*” within the treatment plan.^48^, ^52^ The French Association for Biological Psychiatry and Neuropsychopharmacology (AFPBN) also specifically recommended systematic offering of LAIs as first‐line treatment to schizophrenia patients who needed maintenance treatment.^48^, ^52^

In Hong Kong, the consensus statements on adherence issues in schizophrenia^27^ also stated that “*LAI atypical antipsychotics should be considered as an option among indicated patients early in the course of illness in improving adherence*” and “*Adjunctive psychosocial interventions should be considered to be an integral part of the personalized care package to improve adherence.*” Additionally, the recent recommendations for the optimal care of patients with recent‐onset psychosis developed by experts from the Asia‐Pacific region, Europe, and South Africa^57^ stated that “*LAI APs may play a role in relapse prevention via increased rates of adherence and should be considered as an early‐stage treatment.*” The recommendations also suggested that psychosocial therapies should be used in conjunction with continuous antipsychotic therapy: “*Psychosocial therapies should be implemented early to prevent deterioration of psychosocial and cognitive functions.*”^57^

### 2.9 Advantages and preference of LAI SGAs over LAI FGAs

Compared with the older FGA medications, SGAs have been generally associated with a reduced risk of tardive dyskinesia and treatment‐related extrapyramidal syndrome (EPS) which are common side effects of FGAs.^58^ Indeed, 28 neurotoxicity studies have reported the various destructive effects of haloperidol, one of the prototypic FGAs, on brain tissues,^59^ whereas some of the SGAs are associated with neuroprotective effects.^60^ In a review article, some SGAs have been shown to improve cell survival, enhance neurogenesis, and even prevent or reverse the effects of haloperidol‐induced toxicity.^60^

**FIGURE 3:**
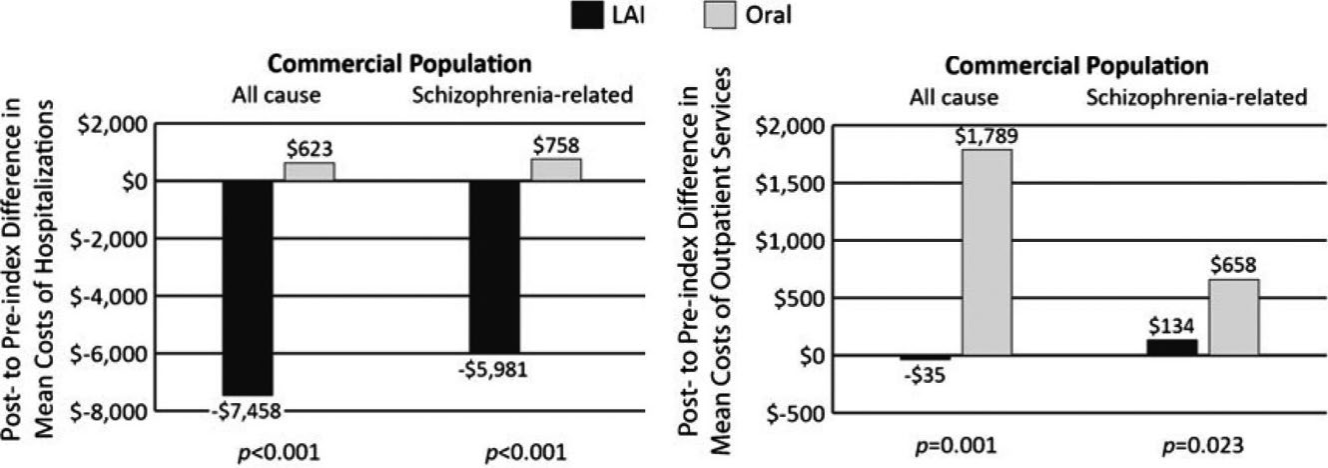
Differences in mean hospitalization and outpatient costs. From Lin et al, 2013^61^

Overall, the advantages of LAI SGAs can be translated to a more cost‐effective treatment strategy. As shown by the results of a real‐world study, the initial drug costs were higher with LAIs, but the subsequent schizophrenia‐related health costs for hospitalizations and outpatient services were higher in patients using oral AP (Figure 3).^61^ This provides indirect evidence that nonadherence is strongly associated with an increased risk of relapses and hospitalizations, and generally greater costs of care.^61^

### 2.10 LAI SGAs‐to‐LAI FGAs ratio

**FIGURE 4:**
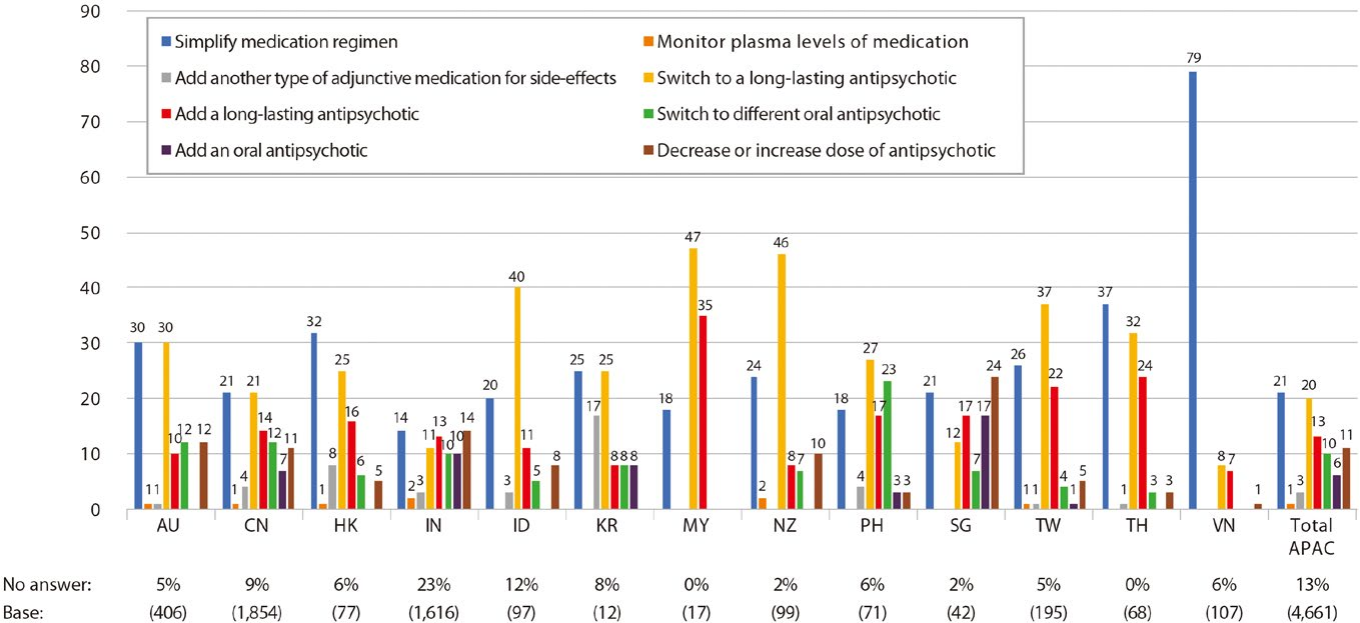
The preferred pharmacological approach for psychiatrists in 13 Asia‐Pacific countries to address an adherence problem in their patients. Modified from Olivares et al, 2013.^4^ APAC, Asia–Pacific; AU, Australia; CN, People's Republic of China; HK, Hong Kong; ID, Indonesia; IN, India; KR, Korea; MY, Malaysia; NZ, New Zealand; PH, Philippines; SG, Singapore; TH, Thailand; TW, Taiwan; VN, Vietnam

The advantages of LAI SGAs clearly demonstrate a need to increase its use in place of LAI FGAs or oral AP. Evidence has shown that among the 13 Asia‐Pacific countries, one in three psychiatrists (33%) expressed preference to switch to or add on a LAI.^4^ Indeed, 41% of the psychiatrists in Hong Kong showed the same preference for addressing an adherence problem in their patients (Figure 4).^4^ An increase in ratio of LAI SGAs‐to‐LAI FGAs would presumably provide many beneficial treatment outcomes to patients.

## 3 CONCLUSION

In light of the advantages of LAI SGAs over both oral AP and LAI FGAs described in this article, the HKAPR panel would like to provide directions for Hong Kong on improving the SMI management for schizophrenia patients within the near future. The proposed recommendations include the implementation of shared decision‐making programs, as well as the increase in LAI SGA‐to‐FGA ratio as described above. The enhancement of LAI SGAs would significantly lead to better drug adherence for patients, leading to better clinical and psychosocial treatment outcomes, not to mention that neurotoxicity and side effects in patients would be significantly decreased. Also, due to better medication adherence, the implementation of LAI SGAs would reduce the rate of relapse and treatment discontinuation, thus increasing the percentage of remission. Additionally, the increase in ratio of LAI SGAs would help reduce the social burden of patients because a decrease in relapses would reduce brain tissue damage. Considering all these factors, the schizophrenia‐related healthcare costs for hospitalizations and outpatient visits would be decreased. The reduction of hospitalization and relapse rates would therefore result in workload reduction for healthcare workers, who could then better align their objectives toward effective psychosocial treatment and reintegrating patients into the community. Ultimately, the augmentation of LAI SGAs would lead to a higher quality of care for patients as a result of better treatment adherence.

## CONFLICT OF INTEREST

The authors declare no conflict of interest.

## References

[1] Hospital Authority . Hospital Authority Mental Health Service Plan for Adults 2010–2015. Hong Kong, China: Hospital Authority; 2009.

[2] Department of Health . Fourth National Mental Health Plan – An agenda for collaborative government action in mental health 2009‐2014. Canberra, Commonwealth of Australia: Department of Health; 2009.

[3] Mental Health Network . The NHS Confederation. Fact sheet: Key Facts and Trends In Mental Health. London, UK: Mental Health Network; 2009.

[4] Olivares JM , Thirunavukarasu M , Kulkarni J , Zhang HY , Zhang M , Zhang F . Psychiatrists' awareness of partial and nonadherence to antipsychotic medication in schizophrenia: results from an Asia‐Pacific survey. Neuropsychiatr Dis Treat. 2013;9:1163‐1170. 23976858 10.2147/NDT.S49080 PMC3747021

[5] Fleischhacker WW , Miyamoto S . Pharmacological treatment of schizophrenia: current issues and future perspectives. Clin Neuropsychopharmacol Ther. 2016;7:1‐8.

[6] Medalia A , Saperstein AM , Hansen MC , Lee S . Personalized treatment for cognitive dysfunction in individuals with schizophrenia spectrum disorders. Neuropsychol Rehabil. 2016;24:1‐12. 10.1080/09602011.2016.1189341 PMC5951177 27219068

[7] Välimäki M , Athanasopoulou C , Lahti M , Adams CE . Effectiveness of social media interventions for people with schizophrenia: a systematic review and meta‐analysis. J Med Internet Res. 2016;18:e92. 27105939 10.2196/jmir.5385 PMC4859871

[8] Canas F , Moller HJ . Long‐acting atypical injectable antipsychotics in the treatment of schizophrenia: safety and tolerability review. Expert Opin Drug Saf. 2010;9:683‐697. 20690885 10.1517/14740338.2010.506712

[9] Cheung EFC , Lam LCW , Hung SF . Hong Kong In: Ghodse H , ed. International Perspectives on Mental Health. London, UK: RCPsych Publications; 2011:96‐100.

[10] Häfner H , an der Heiden W , Behrens S , et al. Causes and consequences of the gender difference in age at onset of schizophrenia. Schizophr Bull. 1998;24:99‐113. 9502549 10.1093/oxfordjournals.schbul.a033317

[11] Alptekin K , Erkoc S , Gogus AK , et al. Disability in schizophrenia: clinical correlates and prediction over 1‐year follow‐up. Psychiatry Res. 2005;135:103‐111. 15923043 10.1016/j.psychres.2004.05.027

[12] van Haren NE , Hulshoff Pol HE , Schnack HG , et al. Focal gray matter changes in schizophrenia across the course of the illness: a 5‐year follow‐up study. Neuropsychopharmacology. 2007;32:2057‐2066. 17327887 10.1038/sj.npp.1301347

[13] Andreasen NC , Liu D , Ziebell S , Vora A , Ho B‐C . Relapse duration, treatment intensity, and brain tissue loss in schizophrenia: a prospective longitudinal MRI study. Am J Psychiatry. 2013;170:609‐615. 23558429 10.1176/appi.ajp.2013.12050674 PMC3835590

[14] Caroli F , Raymondet P , Izard I , Plas J , Gall B , Delgado A . Opinions of French patients with schizophrenia regarding injectable medication. Patient Prefer Adherence. 2011;21:165‐171. 10.2147/PPA.S15337 PMC3090377 21573047

[15] Potkin S , Bera R , Zubek D , Lau G . Patient and prescriber perspectives on long‐acting injectable (LAI) antipsychotics and analysis of in‐office discussion regarding LAI treatment for schizophrenia. BMC Psychiatry. 2013;13:261‐271. 24131801 10.1186/1471-244X-13-261 PMC3819472

[16] Chien WT , Chan SWC , Yeung FKK , Chiu HFK , Ng BFL . Perceived stigmatization of patients with mental illness and its psychosocial correlates: a prospective cohort study. Hong Kong Med J. 2015;21(Suppl 2):27‐31. 25852099

[17] Leamy M , Bird V , Boutillier CL , Williams J , Slade M . Conceptual framework for personal recovery in mental health: systematic review and narrative synthesis. Br J Psychiatry. 2011;199:445‐452. 22130746 10.1192/bjp.bp.110.083733

[18] Slade M , Amering M , Farkas M , et al. Uses and abuses of recovery: implementing recovery‐oriented practices in mental health systems. World Psychiatry. 2014;13:12‐20. 24497237 10.1002/wps.20084 PMC3918008

[19] Slade M . Implementing shared decision making in routine mental health care. World Psychiatry. 2017;16(2):146‐153. 28498575 10.1002/wps.20412 PMC5428178

[20] Wong DFK , Lam AYK , Chan SK , Chan SF . Quality of life of caregivers with relatives suffering from mental illness in Hong Kong: roles of caregiver characteristics, caregiving burdens and satisfaction with psychiatric services. Health Qual Life Outcomes. 2012;10:15‐23. 22289443 10.1186/1477-7525-10-15 PMC3293083

[21] Bressington D , Mui J , Wells H . The effects of medication‐management training on clinicians' understanding and clinical practice in Hong Kong. Nurse Educ Today. 2013;33:969‐975. 23182892 10.1016/j.nedt.2012.10.021

[22] Stevens GL , Dawson G , Zummo J . Clinical benefits and impact of early use of long‐acting injectable antipsychotics for schizophrenia. Early Interv Psychiatry. 2016;10:365‐377. 26403538 10.1111/eip.12278 PMC5054869

[23] Patel MX , De Zoysa N , Baker D , David AS . Antipsychotic depot medication and attitudes of community psychiatric nurses. J Psychiatr Ment Health Nurs. 2005;12:237‐244. 15788043 10.1111/j.1365-2850.2004.00826.x

[24] Patel MX , Yeung FK , Haddad PM , David AS . Psychiatric nurses’ attitudes to antipsychotic depots in Hong Kong and comparison with London. J Psychiatr Ment Health Nurs. 2008;15:758‐766. 18844802 10.1111/j.1365-2850.2008.01306.x

[25] Emsley R , Alptekin K , Azorin J‐M , et al. Nurses’ perceptions of medication adherence in schizophrenia: results of the ADHES cross‐sectional questionnaire survey. Ther Adv Psychopharmacol. 2015;5:339‐350. 26834967 10.1177/2045125315612013 PMC4722504

[26] Patel KR , Cherian J , Gohil K , Atkinson D . Schizophrenia: overview and treatment options. P T. 2014;39:638‐645. 25210417 PMC4159061

[27] Mak KY , Lo WT , Yeung WS , et al. Consensus statements on adherence issues in schizophrenia for Hong Kong. Asian J Psychiatr. 2014;12:163‐169. 25440570 10.1016/j.ajp.2014.06.018

[28] Cheung HK . Schizophrenics fully remitted on neuroleptics for 3–5 years‐to stop or continue drugs? Br J Psychiatry. 1981;138:490‐494. 6117347 10.1192/bjp.138.6.490

[29] Chen EYH , Hui CLM , Lam MML , et al. Maintenance treatment with quetiapine versus discontinuation after one year of treatment in patients with remitted first episode psychosis: randomised controlled trial. BMJ. 2010;19:c4024. 10.1136/bmj.c4024 PMC2924475 20724402

[30] Chui WWH , Mui JHC , Cheng KM , Cheung EFC . Community psychiatric service in Hong Kong: moving towards recovery‐oriented personalized care. Asia Pac Psychiatry. 2012;4:155‐159.

[31] Glazer WM . Who receives long‐acting antipsychotic medications? Psychiatr Serv. 2007;58:437. 17412839 10.1176/ps.2007.58.4.437

[32] Jimmy B , Jose J . Patient medication adherence: measures in daily practice. Oman Med J. 2011;26:155‐159. 22043406 10.5001/omj.2011.38 PMC3191684

[33] Lieberman JA , Alvir JM , Koreen A , et al. Psychobiologic correlates of treatment response in schizophrenia. Neuropsychopharmacology. 1996;14(Suppl 3):13S‐21S. 8866739 10.1016/0893-133X(95)00200-W

[34] Kane JM . Treatment adherence and long‐term outcomes. CNS Spectr. 2007;12(Suppl 17):21‐26. 17934386 10.1017/s1092852900026304

[35] Kane JM . Treatment strategies to prevent relapse and encourage remission. J Clin Psychiatry. 2007;68(Suppl 14):27‐30. 18284275

[36] Soundy A , Stubbs B , Roskell C , Williams SE , Fox A , Vancampfort D . Identifying the facilitators and processes which influence recovery in individuals with schizophrenia: a systematic review and thematic synthesis. J Ment Health. 2015;24:103‐110. 25643043 10.3109/09638237.2014.998811

[37] van Langen WJM , Beentjes TAA , van Gaal BGI , Nijhuis‐van der Sanden Maria WG , Goossens PJJ . How the illness management and recovery program enhanced recovery of persons with schizophrenia and other psychotic disorders: a qualitative study. Arch Psychiatr Nurs. 2016;30:552‐557. 27654236 10.1016/j.apnu.2016.04.005

[38] Robinson D , Woerner MG , Alvir JM , et al. Predictors of relapse following response from a first episode of schizophrenia or schizoaffective disorder. Arch Gen Psychiatry. 1999;53:241‐247. 10.1001/archpsyc.56.3.241 10078501

[39] Wiersma D , Nienhuis FJ , Slooff CJ , Giel R . Natural course of schizophrenia disorders: a 15‐year follow up of a Dutch incidence cohort. Schizophr Bull. 1998;24:75‐85. 9502547 10.1093/oxfordjournals.schbul.a033315

[40] Hogan TP , Awad AG , Eastwood R . A self‐report scale predictive of drug compliance in schizophrenics: reliability and discriminative validity. Psychol Med. 1983;13:177‐183. 6133297 10.1017/s0033291700050182

[41] Thompson K , Kulkarni J , Sergejew AA . Reliability and validity of a new medication adherence rating scale (MARS) for the psychoses. Schizophr Res. 2000;42:241‐247. 10785582 10.1016/s0920-9964(99)00130-9

[42] Byerly MJ , Nakonezny PA , Rush AJ . The brief adherence rating scale medication adherence of outpatients with schizophrenia and schizoaffective disorder. Schizophr Res. 2008;100:60‐69. 18255269 10.1016/j.schres.2007.12.470

[43] Cushing A , Metcalfe R . Optimizing medicines management: from compliance to concordance. Ther Clin Risk Manag. 2007;3:1047‐1058. 18516274 PMC2387303

[44] Jordan JL , Ellis SJ , Chambers R . Defining shared decision making and concordance: are they one and the same? Postgrad Med J. 2002;78:383‐384. 12151651 10.1136/pmj.78.921.383 PMC1742432

[45] Elwyn G , Frosch D , Thomson R , et al. Shared decision making: a model for clinical practice. J Gen Intern Med. 2012;27:1361‐1367. 22618581 10.1007/s11606-012-2077-6 PMC3445676

[46] South China Morning Post , October 2012. http://www.scmp.com/news/hong-kong/article/1054859/one-three-suffer-mental-illness-survey-finds. Accessed September 27, 2016.

[47] South China Morning Post , December 2012. http://www.scmp.com/news/hong-kong/article/1098943/long-working-hours-cause-depression-survey-confirms. Accessed July 11, 2016.

[48] Kim B , Lee SH , Yang YK , et al. Long‐acting injectable antipsychotics for first‐episode schizophrenia: the pros and cons. Schizophr Res Treatment. 2012;2012:560836. 22966439 10.1155/2012/560836 PMC3425805

[49] Masand PS , Roca M , Turner MS , Kane JM . Partial adherence to antipsychotic medication impacts the course of illness in patients with schizophrenia: a review. Prim Care Companion J Clin Psychiatry. 2009;11:147‐154. 19750066 10.4088/PCC.08r00612 PMC2736032

[50] Haddad PM , Brain C , Scott J , et al. Nonadherence with antipsychotic medication in schizophrenia: challenges and management strategies. Patient Relat Outcome Meas. 2014;5:43‐62. 25061342 10.2147/PROM.S42735 PMC4085309

[51] Fe Bravo‐Ortiz M , Gutiérrez‐Casares JR , Rodríguez‐Morales A , et al. Influence of type of treatment on the well‐being of Spanish patients with schizophrenia and their caregivers. Int J Psychiatry Clin Pract. 2011;15:286‐295. 22122003 10.3109/13651501.2011.608469

[52] Heres S , Lambert M , Vauth R . Treatment of early episode in patients with schizophrenia: the role of long acting antipsychotics. Eur Psychiatry. 2014;29(Suppl 2):1409‐1413. 25455704 10.1016/S0924-9338(14)70001-X

[53] Lafeuille MH , Laliberté‐Auger F , Lefebvre P , et al. Impact of atypical long‐acting injectable versus oral antipsychotics on rehospitalization rates and emergency room visits among relapsed schizophrenia patients: a retrospective database analysis. BMC Psychiatry. 2013;13:221. 24016390 10.1186/1471-244X-13-221 PMC3847215

[54] Warner R . Recovery from schizophrenia and the recovery model. Curr Opin Psychiatry. 2009;22:374‐380. 19417668 10.1097/YCO.0b013e32832c920b

[55] Chien WT , Leung SF , Yeung FKK , et al. Current approaches to treatments for schizophrenia spectrum disorders, part II: psychosocial interventions and patient‐focused perspectives in psychiatric care. Neuropsychiatr Dis Treat. 2013;9:1463‐1481. 24109184 10.2147/NDT.S49263 PMC3792827

[56] Valencia M , Juarez F , Ortega H . Integrated treatment to achieve functional recovery for first‐episode psychosis. Schizophr Res Treatment. 2012;2012:962371. 22970366 10.1155/2012/962371 PMC3420493

[57] Lo TL , Warden M , He Y , et al. Recommendations for the optimal care of patients with recent‐onset psychosis in the Asia‐Pacific region. Asia Pac Psychiatry. 2016;8:154‐171. 27062665 10.1111/appy.12234 PMC4834614

[58] Gopal S , Berwaerts J , Nuamah I , et al. Number needed to treat and number needed to harm with paliperidone palmitate relative to long‐acting haloperidol, bromperidol, and fluphenazine decanoate for treatment of patients with schizophrenia. Neuropsychiatr Dis Treat. 2011;7:93‐101. 21552311 10.2147/NDT.S17177 PMC3083982

[59] Nasrallah HA . Haloperidol clearly is neurotoxic. Should it be banned? Current Psychiatry. 2013;12:7‐8.

[60] Nandra KS , Agius M . The differences between typical and atypical antipsychotics: the effects on neurogenesis. Psychiatr Danub. 2012;24(Suppl 1):S95‐S99. 22945197

[61] Lin J , Wong B , Offord S , Mirski D . Healthcare cost reductions associated with the use of LAI formulations of antipsychotic medications versus oral among patients with schizophrenia. J Behav Health Serv Res. 2013;40:355‐366. 23579871 10.1007/s11414-013-9329-z

