# Corrigendum

**DOI:** 10.1111/cns.13721

**Published:** 2021-08-19

In Lo et al.,^1^ the name of the first author was misspelled in the published version. The correct name should be presented as ‘William Tak‐Lam Lo’.
